# Other PHAC publications

**DOI:** 10.24095/hpcdp.45.2.05

**Published:** 2025-02

**Researchers from the Public Health Agency of Canada also contribute to work published in other journals and books. Look for the following articles published in 2024:**

Biswas A, **Prince SA**. Does remote work promote exercise and cardiovascular health? Current evidence and future directions. Can J Cardiol. 2024. https://doi.org/10.1016/j.cjca.2024.09.029

**Dopko R, Liu L, Contreras G**. Suicidal ideation and mental illnesses during the COVID-19 pandemic in Canada. Discov Psychol. 2024;4(1):151. https://doi.org/10.1007/s44202-024-00253-z

**Hyun Lim S, Hersi M, Krishnan R, Montroy J, Rook B, Farrah K, Chung Y, Stevens A, Zafack J, Wong E, Forbes N, Killikelly A, Young K, Tunis M.** COVID-19 vaccine evidence monitoring assisted by artificial intelligence: an emergency system implemented by the Public Health Agency of Canada to capture and describe the trajectory of evolving pandemic vaccine literature. Vaccine X. 2024;21:100575. https://doi.org/10.1016/j.jvacx.2024.100575

John-Baptiste A, Moulin M, Li Z, Hamilton D, Crichlow G, Klein DE, […] **Moqueet N, Champredon D**, et al. Do COVID-19 infectious disease models incorporate the social determinants of health? A systematic review. Public Health Rev. 2024;45:1607057. https://doi.org/10.3389/phrs.2024.1607057

**Robinson J**, Chaput JP, **Roberts KC**, Goldfield GS, **Wong SL**, Janssen I, **Garipy G, Prince SA, Capaldi CA, Lang J**. Sleep health characteristics and positive mental health in Canadian youth: a cross-sectional analysis of the Health Behaviour in School-aged Children study. Sleep Health. 2024;10(6):671-7. https://doi.org/10.1016/j.sleh.2024.09.008

Sprague AE, Roberts NF, Lavin Venegas C, Nath T, Shah PS, Barrett J, […] **Dzakpasu S**, et al. Mortality following childbirth in Ontario: a 20-year analysis of temporal trends and causes. J Obstet Gynaecol Can. 2024;46(12):102689. https://doi.org/10.1016/j.jogc.2024.102689

Yin CY, **Scott MM**, Kimura M, Hakimjavadi R, Girard CI, Clarke A, et al. Oral anticoagulant use and post-fall mortality in long-term care home residents. J Am Med Dir Assoc. 2024;25(12):105233. https://doi.org/10.1016/j.jamda.2024.105233

**Zakaria D, Demers A, Cheta N, Aziz S, Abdullah P**. Factors associated with the development, severity, and resolution of post COVID-19 condition in adults living in Canada, January 2020 to August 2022. Can J Public Health. 2024. https://doi.org/10.17269/s41997-024-00958-7

